# Pregnancy after Double Oophorectomy

**Published:** 1892-06

**Authors:** 


PREGNANCY AFTER DOUBLE OOPHORECTOMY.—Dr. J. A Robertson ("Annals of Gyn. and Pæd.") has performed double oopho. rectomy for cystic ovarian disease in a 23-year-old girl- Three months thereafter she menstruated. Five months after she married, became pregnant and was delivered of a healthy boy.—(*Ex.*)

